# Recurrent Febrile Neutropenia and Thrombocytopenia in a Chronic Cocaine User: A Case of Levamisole Induced Complications

**DOI:** 10.1155/2015/303098

**Published:** 2015-03-22

**Authors:** Eduardo Martinez, Raza Alvi, Sindhaghatta Venkatram, Gilda Diaz-Fuentes

**Affiliations:** ^1^Division of Pulmonary and Critical Care Medicine, Bronx Lebanon Hospital Center, Bronx, NY 10457, USA; ^2^Albert Einstein College of Medicine, Bronx, NY 10461, USA; ^3^Department of Internal Medicine, Bronx Lebanon Hospital Center, Bronx, NY 10457, USA

## Abstract

Cocaine is used by approximately 1.5 million Americans each month and up to 69% of the cocaine seized contains levamisole. The real incidence of cocaine-levamisole induced neutropenia is unclear but probably underestimated. Associated complications include fever, thrombocytopenia, skin-vasculitis disorders, and rarely kidney injury. We present a young male, with chronic active cocaine use presenting with recurrent episodes of febrile neutropenia and thrombocytopenia. He underwent extensive work-up and was treated with many antibiotics and we suspect that his neutropenia and thrombocytopenia were caused by recurrent cocaine-levamisole use.

## 1. Introduction

The etiology for neutropenia is extensive and includes medications, sepsis, and hematological or oncological conditions. Neutropenia has been well described as one of the common side effects of levamisole, an imidazothiazole previously used as an anthelminthic and adjuvant to 5-fluorouracil (5-FU) in the treatment of colon cancer [1]. Due to its serious adverse effects, it was discontinued in the year 2000 [2]. In the past decade there has been resurgence in the use of levamisole, not as a prescribed medication but as an adulterant of up to 69% of the cocaine found in the United States [3]. Analysis of street samples of cocaine has shown an average purity rate of 50%. Average purities fell substantially from 2004 to 2010, decreasing from 69.1% to 50.5% for crack cocaine and from 65.2% to 37.0% for powder cocaine [4]. Therefore, adulterants represent more than half of the composition of all cocaine sold. Adulterants are added to cocaine to promote the perceived potency of the drug or to increase the volume of the drug [5]. Drug dealers may respond to changing drug market and law enforcement pressure by manipulating cocaine quality using adulterants rather than adjusting prices. Adulterants are pharmacologically active substances that are intentionally added to cocaine in order to potentiate its effect. Levamisole, along with its metabolite aminorex, has been proven to enhance noradrenergic neurotransmission by inhibiting reuptake, by acting on ganglionic nicotinic receptors, and by increasing the concentration of endogenous opiate compounds [6, 7]. Levamisole also shares very similar chemical properties with cocaine, such as color and melting point, which makes it almost imposible to distinguish between the two [8]. The fact that patients not only deny the use of cocaine but also are unaware of this combination of drugs makes the diagnosis of cocaine-levamisole associated neutropenia very difficult.

This is relevant to physicians in New York where in 2011, in a yearly survey distributed by the Substance Abuse and Mental Health Services Administration, 2.24% of persons aged 18 years or older stated that they had used cocaine within the past year (3rd behind Rhode Island and Colorado) [9].

Cocaine-levamisole associated neutropenia is frequently self-limited and usually resolves after withdrawing the use of the contaminated cocaine but tends to recur with reexposure [1, 2]. Another adverse effect with similar presentation is thrombocytopenia. This associated thrombocytopenia and the recurrent episodes are seldom reported in the literature.

We present a patient with recurrent episodes of febrile neutropenia and thrombocytopenia which improved after discontinuing the use of cocaine.

## 2. Case Report

A 36-year-old man was admitted to the intensive care unit with fever and right gluteal pain and swelling of three-day duration. He denied trauma, rash, flu-like symptoms, or sick contacts. Medical history included continuous cocaine abuse (sniffing), paroxysmal atrial fibrillation, and two episodes of febrile neutropenia in the past.

On examination, the patient was awake, alert, comfortable, febrile 103.1 F, and tachycardic (120 bpm) nontoxic looking. An abscess was found on the gluteal area. The rest of the skin was intact and the rest of the exam was unremarkable. Laboratory findings showed severe leucopenia with neutropenia and thrombocytopenia. Urine toxicology by immunoassay was reported to be positive for cocaine and cannabinoids.

He was managed for severe sepsis and febrile neutropenia with drainage of his gluteal abscess, fluids, broad spectrum antibiotics, including caspofungin, and granulocyte colony-stimulating factor (G-CSF). Serum and urine levamisole levels performed by high performance liquid chromatography/tandem mass spectrometry (LC-MS/MS) five days after admission were negative. Flow cytometry, cultures, serology, HIV, and vasculitis work-up were done and ruled out other common causes of neutropenia and thrombocytopenia (Table 1). Despite high fevers, the patient remained stable and asymptomatic. Clinical course was complicated by acute kidney injury on day 5 of admission. Urine analysis revealed no eosinophils and benign sediment.

On review of medical records, the patient had been admitted to our institution twice during the last 12 months for febrile neutropenia. Table 1 shows summary of presentations during all admissions including the current one.

Patient became afebrile on day 7 of admission with resolution of thrombocytopenia and some improvement of WBC. He was discharged home in stable condition and he was lost to follow-up.

## 3. Discussion

Approximately 1.5 million Americans use cocaine each month and, according to a report from the Drug Enforcement Administration (DEA) in July 2009, 69% of the cocaine seized coming into the United States contained levamisole [10]. Levamisole, neutropenia, thrombocytopenia, and other serious side effects associated with levamisole use were first reported in the 1970s when it was used for inflammatory conditions and as an adjuvant treatment of colon and breast cancer [11, 12]. Neutropenia has been reported in 60–69% of cases of cocaine-levamisole induced complications [1, 13].

The pathophysiology of this syndrome is not completely understood, but drugs with reactive thiol groups, such as levamisole, behave as haptens and trigger immune or cytotoxic response, causing opsonization and destruction of white blood cells leading to agranulocytosis [14]. Autoantibody formation and human leukocyte antigen B27 (HLA B27) status have also been proposed [15, 16]. Pure cocaine has recognizable cardiovascular and neurological toxic effects, but there is no evidence that it can cause neutropenia and thrombocytopenia [5, 17].

The most common reported complications are skin involvement with a retiform purpura with or without bullae on the helix of the ears or extremities and a self-limited neutropenia. These can occur as isolated manifestations or simultaneously. Although thrombocytopenia has been reported as an adverse effect of levamisole when used for medical purposes, a literature review of 203 cases by Larocque et al. revealed only 4 (2%) cases with thrombocytopenia. Other less common complications are fever, arthralgias, hyponatremia, and kidney injury. Although recurrence is seen with reexposure to the contaminated cocaine, it is not commonly reported [1, 18]. Our patient presented with febrile neutropenia and thrombocytopenia and developed acute kidney injury (AKI). Lee et al. reported 30 cases of anti-neutrophil cytoplasmic antibodies (ANCA) positivity associated with cocaine ingestion; they all had anti-myeloperoxidase antibodies (MPO) and 50% also had anti-proteinase 3 antibodies. Two of the thirty cases (6.6%) had AKI [18]. It is unclear whether our patient developed AKI due to levamisole or neprotoxicity due to medications and contrast.

**Figure 1:**
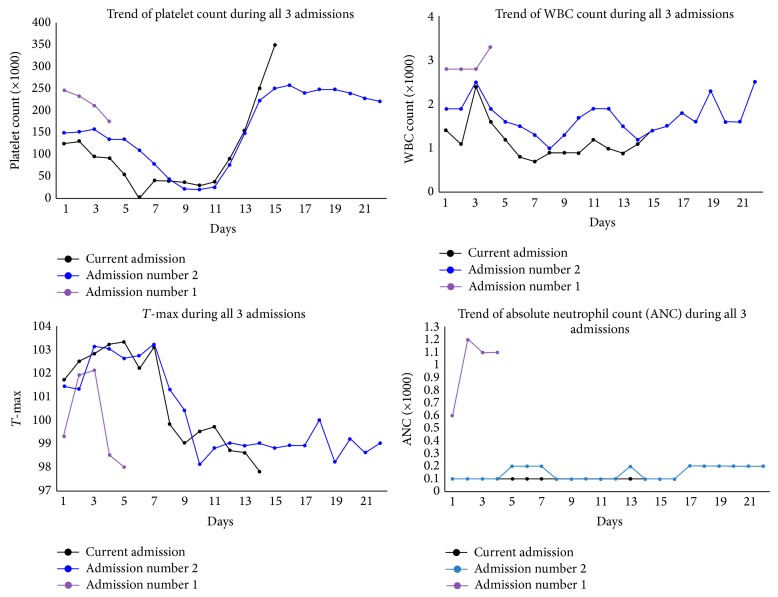
Graph of trend for platelets, temperature, and WBC and ANC count.

On review of all the admissions for our patient, it is interesting to note that the nadir for the thrombocitopenia occured between day 5 and 8 and, similarly, the fever curve improved between days 5 and 8 of presentation with marginal improvement in WBC count (Figure 1).

The diagnosis of cocaine-levamisole complications is a diagnosis of exclusion. It is difficult to distinguish this condition from other forms of vasculitis. A high-titer c-ANCA, p-ANCA, human neutrophil elastase ANCA (HNE-ANCA), with concomitant antinuclear, anti-phospholipid antibodies and 11 isolated skin vasculitis suggests of cocaine-levamisole as etiological agent [11]. The detection of levamisole in the serum or urine assists in the diagnosis, but the absence does not rule out levamisole as the etiology since its half-life is only approximately 5.6 hours [2, 19]. In our patient the vasculitis work-up and the levamisole levels were negative.

Management of the condition is conservative with discontinuation of the levamisole contaminated cocaine and treatment of complications. This usually leads to a rapid clinical improvement in 2-3 weeks. Other modalities such as systemic steroids and G-CSF are also recommended without consensus about their benefits [10, 14]. Use of G-CSG is supportive and suggested for patients with very severe febrile neutropenia with absolute neutrophil count (ANC) less than 0.1 × 10^9^/L. Our patient was initially treated with G-CSF in two of the admissions with no improvement in WBC count.

## 4. Conclusion

Cocaine-levamisole induced febrile neutropenia should be highly suspected in patients presenting with a positive cocaine test and neutropenia, vasculitis, thrombocytopenia, and positive ANCA. A careful review of prior admissions is warranted and we speculate that there is a relationship between improvement in platelets count and fever resolution in those patients.

Awareness of this entity will allow clinicians to early identify serum and urine levamisole levels and, if no readily identifiable source of fever, take these into consideration to avoid extensive and potentially costly and dangerous procedures and medications.

Patients should be advised of possible recurrence as long as they continue using these substances.

## Figures and Tables

**Table 1:** Comparison of characteristics during the three admissions.

	Admission number 1	Admission number 2	Current admission
Presentation	Fever and sore throat	Fever, neck stiffness, cough, macular rash with whitish central papules on chest and extremities	Fever, gluteal abscess

WBC (ANC)	2.8 k/uL (600 cells/uL)	1.9 k/uL (100 cells/uL)	1.3 k/uL (100 cells/uL)

Platelets lowest	176 k/uL	53 k/uL	33 k/uL

Urine toxicology	Cocaine and cannabinoids	Cocaine	Cocaine and cannabinoids

Imaging	Chest X-ray-negative	Chest X-ray-negative	Chest X-ray-negative
	Chest CT negative	CT head-negative	
	Abdomen/pelvis CT-possible colitis	CT facial bones negative	Abdomen/pelvis CT-perianal abscess
		Echocardiogram-normal	Echocardiogram-normal

Serology-negative	HIV-negative Thyroid panel-normal Cardiac markers-negative	HIV, HTLV, BCR-abl, malaria smear, dengue titer, hepatitis panel serum cryptococcal antigen, collagen vascular disease work-up negative, RPR	ANCA, ANA, MPO, antiphospholipid antibodies, anti-cardiolipin, complement, HLA B27, malaria, hepatitis, HIV, HTLV-all negative

Other tests		Flow cytometry neg.	Flow cytometry neg.

Urine and serum for levamisole	Not done	Not done	Sent at day 5 of admission-negative

Cultures-blood, urine, stools	Negative	Negative	Negative

Procedures	None	Spinal tap normal	Perianal abscess drainage at the bed side
		Bone marrow biopsy ×2 (hypercellular marrow with myeloid predominance and trilineage maturation)	

Antibiotics	Ciprofloxacin Metronidazole Moxifloxacin	Vancomycin, meropenem, acyclovir, fluconazole, doxycycline, daptomycin, metronidazole, cefepime, clindamycin	Vancomycin, cefepime, Zosyn, caspofungin, amikacin, and meropenem

G-CSF (duration-days)	Not given	5 days of administration	8 days of administration

Others			Platelets transfusion

Hospital complications	None	None	Acute renal failure. Serum Creatinine 1.4 → 2.9 → 4.4
			Gastrointestinal bleeding

Fever resolution-time days	2 days	8 days	7 days

Outcome	Discharged after 4 days-symptoms improved	Discharged after 23 days-symptoms improved	Discharged after 16 days-symptoms improved, abscess healing

ANA = antinuclear antibodies; ANCA = antineutrophil cytoplasmic antibodies; HIV = human immunodeficiency virus; HTLV = human T-lymphotropic virus; MPO = anti-myeloperoxidase; human leukocyte antigen B27.
